# Effects of severe obstetric complications on women’s health and infant mortality in Benin

**DOI:** 10.1111/j.1365-3156.2010.02534.x

**Published:** 2010-04-08

**Authors:** Véronique Filippi, Sourou Goufodji, Charalambos Sismanidis, Lydie Kanhonou, Edward Fottrell, Carine Ronsmans, Eusèbe Alihonou, Vikram Patel

**Affiliations:** 1 Department of Epidemiology and Population Health, London School of Hygiene and Tropical MedicineLondon, UK; 2 Centre de Recherche en Reproduction Humaine et en DémographieCotonou, Benin; 3 Umeå Centre for Global Health Research, Umeå UniversityUmeå, Sweden

**Keywords:** obstetric labour complications, pregnancy complications, adverse effects, infant mortality, post-partum period

## Abstract

**Objective:**

To document the impact of severe obstetric complications on post-partum health in mothers and mortality in babies over 12 months in Benin and to assess whether severe complications associated with perinatal death are particularly likely to lead to adverse health consequences.

**Methods:**

Cohort study which followed women and their babies after a severe complication or an uncomplicated childbirth. Women were selected in hospitals and interviewed at home at discharge, and at 6 and 12 months post-partum. Women were invited for a medical check-up at 6 months and 12 months.

**Results:**

The cohort includes 205 women with severe complications and a live birth, 64 women with severe complications and perinatal death and 440 women with uncomplicated delivery. Women with severe complications and a live birth were not dissimilar to women with a normal delivery in terms of post-partum health, except for hypertension [adjusted OR = 5.8 (1.9–17.0)], fever [adjusted OR = 1.71 (1.1–2.8)] and infant mortality [adjusted OR = 11.0 (0.8–158.2)]. Women with complications and perinatal death were at increased risk of depression [adjusted OR = 3.4 (1.3–9.0)], urine leakages [adjusted OR = 2.7 (1.2–5.8)], and to report poor health [adjusted OR = 5.27 (2.2–12.4)] and pregnancy’s negative effects on their life [adjusted OR = 4.11 (1.9–9.0)]. Uptake of post-natal services was poor in all groups.

**Conclusion:**

Women in developing countries face a high risk of severe complications during pregnancy and delivery. These can lead to adverse consequences for their own health and that of their offspring. Resources are needed to ensure that pregnant women receive adequate care before, during and after discharge from hospital. Near-miss women with a perinatal death appear a particularly high-risk group.

## Introduction

Severe obstetric complications are common in low-income countries. Lack of accessible, good quality emergency services means that many women who suffer acute complications do not receive the care they need promptly and become critically ill (‘near-miss’) or die from the complications. Their babies may also suffer impairment or death. Recognition is growing that severe obstetric complications might lead to long-term adverse consequences for women’s health and socio-economic conditions (Gill *et al.* 2007). Yet, post-partum interventions are often disparaged as a means to fight maternal mortality (Campbell & Graham 2006) even though the risk of maternal death remains high up to 6 months after delivery (Hurt *et al.* 2008). Consequently, there are few examples of developing-country research on the health of recently delivered women (Bhatia & Cleland 1996; Fortney & Smith 1996; Uzma *et al.* 1999; Bang *et al.* 2004; Fronczack *et al.* 2005) and more specifically among survivors of complications and their babies, except for one recent study in Burkina Faso (Filippi *et al.* 2007; Storeng *et al.* 2008).

This article describes the impact of near-miss complications on physical and mental health problems in mothers and mortality in babies over 12 months’ follow-up in south Benin. It considers whether, compared to uncomplicated childbirth, any near-miss event is associated with increased adverse outcomes or only those which are associated with perinatal death and discusses the findings implications for post-natal care.

## Methods

### Study setting

Benin is a west African country with a maternal mortality ratio reaching 840 deaths per 100 000 live births in 2005 (Hill *et al.* 2007). The study was conducted in Cotonou, Porto Novo and neighbouring communities in the south of Benin. The proportion of women delivering in health facilities in this area is above 97% according to the 2006 Demographic and Health Survey (INSAE 2007).

### Study design

We conducted a prospective cohort study contrasting the post-partum health of three groups of women who gave birth in six referral facilities over a 12-month period: women with a near-miss complication and live birth; women with a near-miss complication and a stillbirth or perinatal death before discharge; and women with uncomplicated childbirth. Recruitment took place in hospitals to avoid exposure misclassification as it is not possible to determine with accuracy through retrospective interview if a woman has experienced a severe obstetric complication (Stewart & Festin 1995; Ronsmans *et al.* 1997).

### Exposure

Near-miss events were defined in such a way that women would be unlikely to survive without hospital care. They included five categories of complications occurring at term: severe haemorrhage (leading to shock, emergency hysterectomy and/or blood transfusion); hypertensive diseases of pregnancy (BP > 140/90 mmHg or increase in systolic BP > 30 mmHg or diastolic BP > 15 mmHg with at least one other sign of severity such as convulsion or coma); severe dystocia (uterine rupture and Bandl’s ring); severe infections (hyperthermia, hypothermia and/or a clear source of infection with clinical signs of shock); and severe anaemia (haemoglobin level <4 g/dl or 4–7 g/dl or pallor with shock, respiratory difficulties or transfusion). An uncomplicated childbirth was defined as a vaginal birth of a healthy infant, with no deformities, of at least 2500 g at term (37–42 weeks) and with no prenatal, labour or immediate post-partum complications in the mother.

### Outcomes of interest

We measured post-partum health from a broad perspective, including social aspects of health, giving equal weight to women’s self-reports and assessments based on screening and diagnoses tools. We report on perception of physical health; depression and anxiety; reproductive and sexual health; social aspects of health (measured by questions on negative feelings and blame); diagnoses of reproductive morbidity (incontinence, urinary infection) and chronic morbidity that may have been adversely affected by the complications (malnutrition, mild and moderate anaemia, hypertension); as well as use of routine post-natal services and mortality in the infants.

### Recruitment and data collection

Women living within 25–30 km of the hospitals were recruited by eight research midwives between September 2004 and January 2005. This radius was chosen to facilitate good access to women’s homes. In addition, it minimised the differences in socio-economic status between women who had an uncomplicated delivery or a complication, as they came from the same peri-urban area.

The research midwives identified all near-miss cases and a sample of women with uncomplicated births prospectively. They asked the women for consent as soon as they had sufficiently recovered and summarised information from medical records. Whenever possible, they accompanied the women home to locate their addresses.

The research midwives interviewed women at home three times using structured questionnaires: shortly after discharge to evaluate their health status prior to delivery and at 6 and 12 months to measure the health consequences of complications and delivery. The 6- and 12-month questionnaires included WHO’s World Mental Health Survey K10 screening tool designed to detect probable cases of depression (Kessler *et al.* 2002; Baggaley *et al.* 2007).

Women were invited to attend a medical examination with obstetricians, preferably within 1 week of the 6- and 12-month interviews. Anaemia was tested using HemoCue (Hudson-Thomas *et al.* 1994) and urinary tract infections were tested using MacConkey Agar and Cled Medium dip slides. A 45-min Pad test was conducted at 6 months to assess incontinence.

### Funder and ethical clearance

The study was funded by WHO. It was approved by the ethics committees of WHO, the Ministry of Health in Benin and the London School of Hygiene and Tropical Medicine. All women provided informed consent.

### Statistical methods

Regression models were used for each outcome to investigate the effect of near-miss live birth and near-miss perinatal death compared to uncomplicated childbirth. Both unadjusted regression models, including mother group as the only covariate, and adjusted models, for identified important confounders, were fitted. Wealth quintiles were computed using principal components analysis (Filmer & Pritchett 2001). We used logistic regression to obtain the adjusted odd ratios for infant mortality. For the other sequelae, the longitudinal measurement of all outcomes at month 6 and 12 after hospital discharge was considered by using the appropriate random effects generalised linear regression models which calculated the probability of developing a particular health problem over the entire 12-month period (Brown & Prescott 1999). This modelling approach takes into account correlations between repeated measurements of morbidity for the same women. All analyses were conducted using stata 10 (StataCorp 2008).

## Results

There were 205 near-misses with live births, 64 near-misses with perinatal deaths and 440 uncomplicated delivery women included in the cohort. The proportion followed-up by 12 months was 80% overall, and 75% in the near-miss group. A total of 477 (68%) women undertook a medical examination at 6 months, and 457 (65%) at 12 months. Hypertension was the most common cause of near-miss (42%), followed by dystocia (27%), haemorrhage (26%), anaemia (14%) and infection (2%). Overall, 58% of women with a near-miss complication delivered by caesarean section.

Women with a near-miss complication were generally younger (*P*-value = 0.004, Table 1), less educated (*P* < 0.0001), of poorer background (*P* < 0.0001) and more likely to be primiparas (*P* = 0.005) compared to women with uncomplicated delivery.

**1:** Characteristics of participants upon discharge from hospital (potential confounding factors)

		Near-miss women			
Characteristic		With live birth (*n* = 205)	With perinatal death (*n* = 64)	Normal delivery women (*n* = 440)	Chi-square *P*-value
Near-miss diagnosis (obstetric morbidity)†	Anaemia	11.3%	21.9%	N/A	*P* = 0.046
	Hypertension	47.3%	23.4%	N/A	*P* < 0.0001
	Haemorrhage	21.7%	39.1%	N/A	*P* = 0.006
	Dystocia	26.5%	29.7%	N/A	*P* = 0.240
	Infection	2.0%	3.3%	N/A	*P* = 0.764
Mean age (years)		25.8	26.6	27.3	*P* = 0.004*
Gravidity	1	36.1%	31.3%	25.5%	*P* = 0.005
	2–4	49.8%	43.8%	60.4%	
	5+	14.2%	25.0%	14.1%	
Any formal education		62.0%	51.6%	76.5%	*P* < 0.0001
Marital status	Monogamous	77.2%	71.4%	77.0%	*P* = 0.351
	Polygamous	19.4%	22.2%	21.0%	
	Single	3.4%	6.4%	2.1%	
Wealth quintiles	Most poor	29.6%	33.9%	13.7%	*P* < 0.0001
	2	26.6%	21.0%	20.1%	
	3	18.2%	27.4%	16.4%	
	4	14.3%	8.1%	24.4%	
	Least poor	11.3%	9.7%	25.3%	

N/A, not applicable.

* Analysis of variance comparing age means in the three women groups.

† Totals add up to more than 100% because women can have more than one complication.

### Post-partum outcomes for women with a near-miss complication and a live birth

Women with a near-miss live birth were more likely to have fever and had more than 4 times higher odds of being hypertensive compared to those with an uncomplicated delivery (Tables 2 and 3). These women were significantly more likely to have hypertension at 6 and 12 months than other near-miss women. Women with near-miss live birth were not at significantly increased risk of any other physical or psychological diagnoses nor of reporting poorer health status or social aspects of health.

**Table 2:** Findings from clinical examinations at 6 and 12 months after end of pregnancy; post-partum diagnoses outcomes

	Month 6 clinical examination			Month 12 clinical examination		
Data are: n*/N† (%, 95%CI)	Uncomplicated pregnancy	Near-miss live birth	Near-miss perinatal death	Uncomplicated pregnancy	Near-miss live birth	Near-miss perinatal death
Maternal health						
Mild, moderate or severe anaemia‡	40/299 (13%, 10–18)	25/134 (19%, 12–26)	14/41 (34%, 20–51)	75/278 (27%, 22–33)	38/127 (30%, 22–39)	14/40 (35%, 21–52)
Proportion underweight (BMI <18 kg/m^2^)	19/293 (6%, 4–10)	8/132 (6%, 3–12)	1/41 (2%, 0.1–13)	21/279 (8%, 5–11)	9/132 (7%, 3–13)	1/39 (3%, 0.1–13)
Hypertension§	18/297 (6%, 4–9)	18/135 (13%, 8–20)	3/41 (7%, 2–20)	2/283 (1%, 0.1–3)	7/131 (5%, 2–11)	3/40 (8%, 2–20)
Urinary infection	29/296 (10%, 7–14)	13/136 (10%, 5–16)	6/40 (15%, 6–30)	62/284 (22%, 17–27)	27/131 (21%, 14–29)	11/40 (28%, 15–44)
Urinary incontinence¶	25/296 (8%, 6–12)	9/132 (7%, 3–13)	5/40 (13%, 4–27)	8/278 (3%, 1–6)	7/129 (5%, 2–11)	5/40 (13%, 4–27)
Fever (≥37.5 °C)	34/295 (12%, 8–16)	26/133 (20%, 13–27)	4/41 (10%, 3–23)	32/280 (11%, 8–16)	16/130 (12%, 7–19)	7/39 (18%, 8–34)

* Total number of women with the event.

† Total number of women undergone clinical examination.

‡ Mild, moderate or severe anaemia defined as ≤11.0 g/dl.

§ Hypertension defined as systolic blood pressure over 140 mmHg and diastolic over 90 mmHg.

¶ Loss of urine when patient coughs or a weight gain cut-off for the PAD test of >1 g (comparing weight after–before the test) which indicates high risk of incontinence.

**Table 3:** Crude and adjusted odd ratios (OR) and 95% confidence intervals (95% CI) for each near-miss group compared to women with normal delivery (reference category)

	Unadjusted			Adjusted†		
OR (95% CI) *P*-value*	Near-miss live birth	Near-miss perinatal death	LRT test‡	Near-miss live birth	Near-miss perinatal death	LRT test§
Maternal health						
Anaemia¶	1.35 (0.83–2.20) *P* = 0.23	2.63 (1.26–5.52) *P* = 0.01	0.03	1.23 (0.74–2.04) *P* = 0.42	1.90 (0.88–4.09) *P* = 0.10	0.24
Proportion underweight (BMI <18 kg/m^2^)	0.91 (0.38–2.17) *P* = 0.83	0.28 (0.04–1.83) *P* = 0.18	0.35	0.68 (0.27–1.73) *P* = 0.42	0.27 (0.04–1.83) *P* = 0.18	0.30
Hypertension**	4.31 (1.47–12.68) *P* = 0.01	2.40 (0.51–11.41) *P* = 0.27	0.01	5.77 (1.95–17.04) *P* = 0.002	1.80 (0.34–9.64) *P* = 0.49	0.002
Urinary infection	0.94 (0.60–1.48) *P* = 0.80	1.49 (0.77–2.87) *P* = 0.23	0.45	0.89 (0.56–1.42) *P* = 0.62	1.54 (0.78–3.04) *P* = 0.21	0.34
Urinary incontinence††	1.07 (0.58–1.98) *P* = 0.83	2.34 (1.11–4.96) *P* = 0.03	0.11	1.06 (0.55–2.03) *P* = 0.86	2.66 (1.22–5.83) *P* = 0.01	0.07
Fever (≥37.5 °C)	1.51 (0.95–2.40) *P* = 0.08	1.24 (0.58–2.63) *P* = 0.58	0.22	1.71 (1.06–2.76) *P* = 0.03	1.53 (0.71–3.31) *P* = 0.28	0.08

* Wald test *P*-value from the regression model.

† ORs adjusted for: age, wealth quintiles and education.

‡ Likelihood ratio test assessing whether the model adjusting for women’s groups fits the data better when compared to the null model.

§ Likelihood ratio test assessing whether the model also adjusting for women’s groups fits the data better compared to the model with age, formal education and wealth quintiles.

¶ Mild, moderate or severe anaemia defined as ≤11.0 g/dl.

** Hypertension defined as systolic blood pressure over 140 mmHg and diastolic over 90 mmHg.

†† Loss of urine when patient coughs or a weight gain cut-off for the PAD test of >1 g, which indicates high risk of incontinence.

There were five infant deaths (1.1%) after discharge in the uncomplicated delivery group compared to 14 deaths (6.8%) in the near-miss live birth group at 12 months after delivery (*P* < 0.0001). The odds ratio for infant mortality was 5.3 (1.6–17.3), *P* = 0.006. The adjusted odds ratio (for education, wealth quintiles and age) was 11.0 (0.8–158.2), *P* = 0.08. The adjustment seems to increase the main association but the estimates now display greater imprecision.

### Post-partum outcomes for women with a near-miss complication and a perinatal death

Women with a near-miss perinatal death were more likely to suffer from urinary incontinence compared to women with an uncomplicated delivery (Tables 2 and 3). They were also more likely to have lower haemoglobin levels, but this effect disappears when controlling for confounders, indicating that this increase in anaemia in the perinatal death group was linked to the socio-economic status of the women prior the delivery. They were not at a significantly increased risk for any other diagnoses of a physical problem.

From the self-reported outcomes (Tables 4 and 5), women with a near-miss perinatal death were more likely to feel average/not good/not good at all when compared to uncomplicated delivery mothers. This association remains strong even after adjusting for confounders [adjusted OR 5.27 (2.24–12.40)]. In addition, mothers who experienced a near-miss with perinatal death were at increased risk of depression [adjusted OR 3.42 (1.30–9.01)] compared to women with uncomplicated delivery, while women who had a near-miss with a live birth were not. Another striking, but predictable result is the negative effect the pregnancy had on the woman’s life if she experienced a near-miss event compared to uncomplicated delivery [adjusted OR 1.70 (0.99–2.94)] with live birth and 4.11 [1.87–9.00) for perinatal death]. Women in the perinatal death group were significantly more likely to report pregnancy made them feel blamed by someone else [adjusted OR 9.03 (2.09–39.03)]. Women were also more likely to become pregnant again if they had experienced a near-miss event with perinatal death compared to an uncomplicated delivery (adjusted OR 11.90 (4.89–28.91). Those who experienced pain during sex did not appear to be at increased risk in any particular group. Finally, women with a near-miss event, especially those with a perinatal death, were more likely to use routine post-natal services, but only after adjustment for confounders and with borderline significance.

**Table 4:** Findings from interviews at 6 and 12 months after end of pregnancy; self-reported outcomes

	6 months			12 months		
Data are: n*/N† (%, 95%CI)	Uncomplicated pregnancy	Near-miss live birth	Near-miss perinatal death	Uncomplicated pregnancy	Near-miss live birth	Near-miss perinatal death
Self perceived physical health						
Feeling average/not good/not good at all today	39/390(10%,7–13)	18/17410%, 6–16)	16/5131%, 19–46)	28/3538%, 5–11)	17/15311%, 7–17)	12/4626%, 14–41)
Serious illness	78/38920%, 16–24)	29/17317%, 12–23)	14/5127%, 16–42)	72/35320%, 16–25)	40/15326%, 19–34)	16/4635%, 21–50)
Difficulties in doing physical work‡	99/39025%, 21–30)	34/17420%, 14–26)	15/5129%, 10–33)	72/35121%, 16–25)	31/15320%, 14–28)	9/4620%, 9–34)
Mental health						
Depression –% at or above the K10 14 cut-off score	32/3898%, 6–11)	12/1747%, 4–12)	7/5114%, 6–26)	15/3514%, 2–7)	9/1506%, 3–11)	7/4615%, 6–29)
Most recent suicidal thoughts – within last few months	16/3025%, 3–8)	8/1406%, 2–11)	6/4414%, 5–27)	8/1173%, 39–94)	3/3100%, 29–100)	0/20%, 0–84)
Negative changes/social support/relations						
Pregnancy has had a negative effect on the woman’s life	32/3908%, 6–11)	22/17413%, 8–19)	15/5129%, 17–44)	27/3548%, 5–11)	17/15211%, 7–17)	6/4613%, 5–26)
Pregnancy made her feel blamed by someone close to her	19/3905%, 3–8)	10/1746%, 3–10)	9/5118%, 8–31)	7/3532%, 1–4)	3/1532%, 0.5–6)	5/4611%, 4–24)
Reproductive health						
Dyspareunia	32/24913%, 9–18)	7/789%, 4–18)	4/3711%, 3–25)	3/3061%, 0.2–3)	8/1187%, 3–13)	3/388%, 2–21)
New pregnancy	1/2510.4%, 0.01–2)	0/790%, 0–5)	1/373%, 0.1–14)	12/3164%, 2–7)	2/1172%, 0.2–6)	12/4030%, 17–47)
Contraception use (those having resumed sex since end of pregnancy)	80/25432%, 26–38)	24/8030%, 20–41)	10/3628%, 14–45)	114/31636%, 31–42)	31/12425%, 18–34)	18/4243%, 28–59)
Use of routine post-natal services	151/39039%, 34–44)	65/17437%, 30–45)	25/5149%, 35–63)	Not measured		

* Total number of women with a confirmatory response.

† Total number of women responding to the question.

‡ Difficulty with any of the following tasks: work other than housework e.g. office work, in the fields), fetching wood or water, preparing meals, cleaning, washing clothes, going to the market.

¶The ORs reported for this outcome are from a logistic regression model using a single time-point of measurement at 6 months post hospital discharge.

**Table 5:** Crude and adjusted odd ratios (OR) and 95% confidence intervals (95% CI) for each near-miss group compared to women with uncomplicated delivery (reference category); self-reported outcomes

	Unadjusted			Adjusted†		
OR (95% CI) *P*-value*	Near-miss live birth	Near-miss perinatal death	LRT‡ test	Near-miss live birth	Near-miss perinatal death	LRT§ test
Self-perceived physical health						
Feeling average/not good/not good at all today	1.26 (0.69–2.31) *P* = 0.46	6.90 (2.95–16.16) *P* < 0.0001	< 0.0001	1.17 (0.63–2.18) *P* = 0.61	5.27 (2.24–12.40) *P* < 0.0001	0.0004
Serious Illness	1.06 (0.73–1.56) *P* = 0.75	1.91 (1.07–3.40) *P* = 0.03	0.09	1.00 (0.68–1.48) *P* = 0.99	1.62 (0.90–2.92) *P* = 0.11	0.26
Difficulties in doing physical work	0.78 (0.51–1.21) *P* = 0.27	1.11 (0.56–2.20) *P* = 0.76	0.48	0.92 (0.59–1.44) *P* = 0.73	1.18 (0.59–2.38) *P* = 0.64	0.81
Mental health						
Depression –% at or above the K10 14 cut-off score	1.02 (0.51–2.02) *P* = 0.96	2.97 (1.18–7.50) *P* = 0.02	0.07	1.04 (0.51–2.13) *P* = 0.91	3.42 (1.30–9.01) *P* = 0.01	0.045
Most recent suicidal thoughts – within last few months	1.00 (0.48–2.11) *P* = 0.99	1.81 (0.70–4.69) *P* = 0.22	0.50	0.64 (0.28–1.48) *P* = 0.30	1.44 (0.54–3.88) *P* = 0.47	0.33
Negative changes/social support/relations						
Pregnancy has had a negative effect on the woman’s life	1.69 (1.01–2.85) *P* = 0.048	4.02 (1.92–8.43) *P* < 0.0001	0.001	1.70 (0.99–2.94) *P* = 0.056	4.11 (1.87–9.00) *P* < 0.0001	0.001
Pregnancy made her feel blamed by someone close to her	1.17 (0.42–3.28) *P* = 0.77	10.32 (2.43–43.75) *P* = 0.002	0.001	0.83 (0.27–2.53) *P* = 0.75	9.03 (2.09–39.03) *P* = 0.003	0.002
Reproductive health						
Dyspareunia	1.24 (0.65–2.38) *P* = 0.52	1.55 (0.63–3.79) *P* = 0.34	0.58	1.05 (0.54–2.06) *P* = 0.88	1.45 (0.59–3.59) *P* = 0.42	0.74
New pregnancy	0.44 (0.10–1.96) *P* = 0.28	8.66 (3.84–19.48) *P* < 0.0001	<0.0001	0.53 (0.12–2.42) *P* = 0.41	11.90 (4.89–28.91) *P* < 0.0001	< 0.0001
Contraception use (those having resumed sex since end of pregnancy)	0.55 (0.29–1.04) *P* = 0.07	1.20 (0.48–3.03) *P* = 0.70	0.13	0.76 (0.39–1.46) *P* = 0.41	1.83 (0.70–4.76) *P* = 0.22	0.25
Use of routine post-natal services¶	0.94 (0.65–1.36) *P* = 0.76	1.52 (0.85–2.73) *P* = 0.16	0.32	1.44 (0.95–2.16) *P* = 0.08	2.52 (1.32–4.82) *P* = 0.005	0.01

* Wald test *P*-value from the random effects logistic regression model.

† ORs adjusted for: age, wealth quintiles and education.

‡ Likelihood ratio test assessing whether the model adjusting for women’s groups fits the data better when compared to the null model.

§ Likelihood ratio test assessing whether the model also adjusting for women’s groups fits the data better compared to the model with age, formal education and wealth quintiles.

¶ The ORs reported for this outcome are from a logistic regression model using a single time-point of measurement at 6 months post hospital discharge.

### Maternal mortality post-discharge

There was no significant difference for maternal mortality, with one death in the near-miss group (selected because of severe anaemia and severe hypertension) and one death in the uncomplicated childbirth group (an HIV-positive woman).

## Discussion

Severe obstetric complications led to poor maternal health during a post-partum period extended to 12 months and that the burden was especially large for complications associated with perinatal death. Near-miss women who experienced perinatal death were at greater risk of mental disorder and of reporting poor health compared to women with uncomplicated childbirth. They also felt blamed by others, and had negative feelings towards their pregnancies. Many of these negative experiences are likely to be explained in part by their reduced psychological well-being because of the loss of their baby. At the clinical level, they were also more likely to be diagnosed with urinary incontinence, possibly associated with prolonged labour. Reassuringly, these women reported an increased use of post-natal services compared to normal women, although uptake remained low, at below 50%.

Women with a near-miss who had given birth to a live born baby were not dissimilar to women with uncomplicated childbirth in terms of post-partum perceived or medically documented health status, except for hypertension and infant mortality. The higher risk of post-partum hypertension in these women is a likely consequence of the high number of cases of eclampsia and pre-eclampsia in this group. The higher risk of death in babies born to mothers who experienced a near-miss live birth is of great concern. This finding has programmatic implications as only 37% of the mothers in this group use post-natal services.

Two previous longitudinal studies described the health effects of near-miss complications. Waterstone *et al.* (2003) showed that severe morbidity cases in the UK were more likely to report sexual health problems and increased health services uptake up to 12 months post-partum compared to controls with uncomplicated childbirth. There were no significant differences between the two groups for mental health, but their study did not distinguish effects for the subgroup of near-miss women with perinatal death. The authors explained the sexual health problems reported by some near-miss women by their fear of falling pregnant again, given the traumatic event they had just experienced. In our study, we also found indication that women in the live birth near-miss group were less likely to get pregnant than women in the uncomplicated group, but this was not significant. Fertility remains very high in Benin, with women having an average of just below six children (INSAE 2007) and their social status depends on the number of children they have.

The second study, conducted in Burkina Faso, had a larger sample and an additional comparison group of near-miss women with an early pregnancy loss (Filippi *et al.* 2007). It found a higher maternal mortality post-discharge in the near-miss group, a finding that we could not replicate in our study. Like in Benin, women in the near-miss perinatal death group were more likely to experience mental health problems as expressed by more suicide ideation and babies born alive to mothers who had a near-miss were more likely to die post-discharge. However, near-miss women with early pregnancy loss had the highest self-perceived morbidity; severe anaemia was also significantly higher in that group. The Burkina study also reported that women in the near-miss group expressed more negative feelings and lack of self-esteem up to a year post-partum, that they use services more than the uncomplicated group, but not as much as women would have liked, and that they were under pressure to have another pregnancy quickly. There were therefore similarities in findings between the Burkina Faso and Benin studies, but the perinatal death effect appeared more dramatic in Benin because of size and consistency of the effects across several outcomes.

The psychological distress found in the perinatal death group has been documented in developed countries (Forrest *et al.* 1982; Flenady & Wilson 2008) and is often associated with physical symptoms (Badenhorst & Hughes 2007). The need for increased attention to perinatal mortality has been recognised recently by public health professionals working in developing countries (Martines *et al.* 2005). Debates have so far rightly focussed on the importance of preventing these events. Our findings suggest that these debates should be accompanied by discussions on cost-effective and sustainable approaches to preventing and treating mental health problems among women who experience a perinatal death. It has sometimes been said that women in developing countries are ‘used to’ child mortality, and endure their circumstances (Sheper-Hugues 1992; Finerman 1995). Our study shows that this is not the case, and that efforts should be made to test approaches to prevent prolonged emotional disturbance after perinatal death in all settings (Flenady & Wilson 2008).

The trauma associated with complicated childbirth also has financial consequences. Social insurance is available only to a minority of the population in Benin, and at the time of the study, users of services were often obliged to pay large sums of cash to access emergency care. Because emergency obstetric care and surgery can be particularly expensive, women and their families fall into considerable debt (Borghi *et al.* 2003, 2006). It is possible that women’s negative feelings towards the pregnancy and the feelings that they are being blamed were linked to the economic stress associated with debt and hospital charges (Fottrell *et al.* 2010). Lack of use of services among some women might also be explained by economic stress.

The study used comprehensive measurement tools to ascertain exposure and adverse health outcomes post-partum. The loss-to-follow-up for the interviews was relatively small thanks to procedures adopted to find women in the community. However, it is unlikely that women in the uncomplicated group represent all delivering women in the community. While a very high proportion of women in south Benin give birth in health institutions, many do so in health centres, or private clinics, and not in referral hospitals (INSAE 2007). The women with a near-miss complication, on the other hand, are more likely to represent the larger group in the population because they are unlikely to survive in the community in the absence of specialised hospital care. Adjusting for socio-economic status in the analysis has taken account of some of the differences in background in women with and without a near-miss.

We studied many outcomes, and so increased the probability that a significant result occurred by chance. The excess fever found among near-miss women, for example, may be a chance finding, because it is difficult to explain why a near-miss complication would cause high temperature 6 months after birth.

In addition, the sample may underestimate the incidence or severity of health consequences of near-miss morbidity, because only those women living in relatively close proximity to the recruiting hospitals were included. The consequences for women living in more remote areas with poorer access to health care may differ substantially from those experienced by our sample. Furthermore, there might be measurement bias because the midwife interviewers were not blind to the women’s exposure status.

Our study was primarily designed to document the consequences of maternal obstetric complications, but our findings suggest that perinatal mortality was the most important determinant of poor post-partum health. It would have been useful to have a perinatal death group that had not been exposed to ‘near-miss’, to appreciate whether the adverse effects in the ‘near-miss perinatal death’ group was mostly related to the loss of the baby or to the complications. Given the high level of mortality among the babies of the near-miss women after discharge, it would have also been interesting to assess child morbidity and development, to understand the reasons for this mortality more fully. Finally, we could not look at maternal mortality because of the size of our sample. Higher loss-to-follow up in the near-miss group was explained by younger and uneducated women moving away but could have concealed increased mortality. These limitations call for larger studies with additional comparative groups and end points.

## Implications

The majority of maternal deaths and complications, and by extension their sequelae, are avoidable through skilled birth attendance and emergency obstetric care. However, it is impossible to eliminate near-misses even in the richest settings, and our study shows that some of these women are more likely to be diagnosed or to report health problems at 6 and 12 months post-partum. So, what is the role of post-natal care?

There is a consensus that post-natal care benefits the health of mothers and the survival of babies, and that some groups of women and babies (such as women with HIV or low birth weight babies) will further benefit from targeted interventions because they are particularly at risk of adverse outcomes (Warren *et al.* 2006). Pending results of ongoing clinical trials, there is no agreement about the timing, place, and the staff who should be involved in post-natal care (Warren *et al.* 2006). However, there seems to be little programmatic recognition of the particular difficulties of women who have suffered severe obstetric complications and/or perinatal death. This is partly because the debate on the content of post-natal care in developing countries is driven by the desire to reduce child mortality and much less by the needs to improve maternal health and survival. Our research shows that some of the problems experienced by women and their babies persist beyond the traditional 42 days post-partum period; they were either not identified during routine visits, or were too difficult to resolve. The study also shows the importance of actively reaching women who have lost their babies, as they are essentially ‘forgotten’ by traditional post-natal services (which target the immunisation and growth monitoring of live infants); they may not recognise themselves that many of their post-partum problems are health related and could be reduced with the help of health professionals. There is only one published intervention trial to date that tested the provision of post-natal care in a developing country with a woman-centred approach (Ransjo-Arvidson *et al.* 1998). However, it excluded women with complications. We conclude that women with near-miss complications may benefit from active screening for symptoms or signs of adverse emotional and physical well-being during routine post-natal visits or other contacts with health professionals in the post-partum period. Women who have had a perinatal death or hypertension should be monitored particularly closely and offered additional interventions if needed. We also need a better understanding of the reasons for the relatively low use of post-natal services after a complication and whether outreach services, such as those using community health workers (Bang *et al.* 1999) would be beneficial to women who have had a complication.

## Conclusions

Women in developing countries face a high risk of severe complications during pregnancy and delivery, which in turn adversely affects their own health and the health of their off-spring. Resources to ensure that pregnant women receive adequate routine and emergency care before, during and after discharge from hospital are needed. Near- miss women with a perinatal death appear a particularly high-risk group.
